# Neuropsychological Features of Multiple Sclerosis: Impact and Rehabilitation

**DOI:** 10.1155/2018/4831647

**Published:** 2018-02-27

**Authors:** Lambros Messinis, Panagiotis Papathanasopoulos, Mary H. Kosmidis, Grigorios Nasios, Maria Kambanaros

**Affiliations:** ^1^Neuropsychology Section, Department of Neurology, University of Patras Medical School, 26504 Patras, Greece; ^2^University of Patras Medical School, 26504 Patras, Greece; ^3^Lab of Cognitive Neuroscience, School of Psychology, Aristotle University of Thessaloniki, Thessaloniki, Greece; ^4^Department of Speech and Language Therapy, Higher Educational Institute of Epirus, Ioannina, Greece; ^5^Department of Rehabilitation Sciences, Cyprus University of Technology, Limassol, Cyprus

Dating back to the seminal writings on multiple sclerosis (MS), Charcot's observations of the adverse effects that MS exerts on memory, concept formation, and the intellect [1] were underestimated for many decades in the neurology literature. The medical community, due to the often subtle nature of cognitive deficits in MS and the difficulty in detecting these deficits during routine clinical practice, was initially slow to appreciate them as a core clinical symptom of the disease. Instead, they believed that cognitive impairment was a relatively rare entity in MS, which occurred only in advanced cases with a high level of physical disability and was associated with subcortical dementia [2, 3].

The way the disease was viewed from a neuropsychological perspective, however, changed significantly with the publication of large-scale studies, between 1985 and 1995, that utilized standardized neuropsychological measures and flexible comprehensive test batteries. These studies reported prevalence estimates of between 40 and 65% for cognitive dysfunction in MS, providing evidence that cognitive impairment is a core clinical symptom of MS [4–6].

Cognition is a complex process which, by utilizing cognition individuals, can process information from the environment and through past experiences form behaviors and adaptive strategies. In this sense, a dysfunction of cognition in MS may lead to profound functional limitations, affecting daily functional capacity, vocational activities, and socialization, and may also alter behavior or mood, leading to behavioral disturbances such as aggression or impulsivity and depression or apathy [7, 8]. Cognitive deficits can also affect balance and mobility since impaired attention and distractibility force MS patients to actively think about their walking to reduce potential falls. On the other hand, MS individuals with cognitive decline may limit their social interaction activities fearing apparent forgetfulness, slowness in thinking, or processing information and consequently develop depression. Moreover, they may show decreased compliance with their medication regimen by forgetting to take it or by taking it in the wrong way [9].

Although it is now commonly accepted that roughly one-half of individuals with MS will experience cognitive deficits over the course of the disease, prevalence rates are highly variable and depend to a large extent on the type of MS population studied, the clinical, demographic, and sociodemographic characteristics, and the year conducted [10–12]. In this respect and although MS is traditionally considered a disease of adult onset, with neuropsychological studies in the past focusing exclusively on adults, there are now several studies of pediatric-onset MS that report cognitive impairments in approximately one-third of patients, mainly on motor and mental processing speed, episodic verbal and visuospatial memory, and language (see e.g., [13]).

We now know that persistent and progressive cognitive decline in MS is attributed to a neurodegenerative neuropathological disease process (i.e., diffused axonal damage and brain atrophy). Furthermore, it is well known that white matter (WM) lesions and atrophy contribute substantially to cognitive dysfunction in MS patients, although more recent studies provide evidence that pathological gray matter (GM) lesions may have a significant impact on cognitive functioning [14, 15]. On the other hand, several studies have shown that cognitive impairment is only weakly correlated with physical disability and disease duration [5, 16, 17]. Deficits on measures of information processing speed and episodic memory are the most frequent and robust findings [5, 18], although executive functions are also frequently impaired [18].

As noted previously, these deficits have a multidimensional impact on patients' activities of daily living and should be considered in their treatment and rehabilitation. Although cognitive deficits are prominent and detrimental in MS, incomplete evidence exists as to whether available pharmacological treatment (disease-modifying drugs or symptomatic treatment) might improve or stabilize cognitive deficits [19]. On the contrary, neurobehavioral and neurocognitive interventions have been reported to induce cognitive and behavioral improvements, although their efficiency remains speculative due to methodological variability and lack of ecologically valid outcome measures and investigation over long follow-up periods [20]. More recent studies have successfully applied various neuroimaging techniques (e.g., f-MRI) to study the effects of cognitive rehabilitation in MS. These studies demonstrated an improvement in cognitive functions and everyday functioning capacity by promoting adaptive changes via neuroplasticity in the brains of persons with MS, through the documentation of changes at the level of the cerebral substrate from pre- to posttreatment [21]. Another promising strategy for enhancing cognitive function in MS patients is the use of noninvasive brain stimulation. This includes techniques like repetitive transcranial magnetic stimulation (rTMS), which has recently been proven to be beneficial in MS patients [22].

This special issue entails a series of cutting-edge articles that provide innovative research findings and recent information on the impact and rehabilitation of neuropsychological impairment in MS. Specifically, the authors of this special issue addressed cognitive impairment in relapsing-remitting MS (RRMS) patients that present very mild clinical disability; clinical, neuropsychological, and neuroradiological features in pediatric onset multiple sclerosis; relationships between MS impairment, unmet family needs, and caregiver mental health; the efficacy of computer-assisted cognitive rehabilitation in RRMS patients; a proposed research protocol of an innovative efficacy study on the impact of telestimulation or distance cognitive stimulation in MS; and a review article providing the most recent information and findings on the efficacy of repetitive transcranial magnetic stimulation (rTMS) in alleviating cognitive impairment in MS.

The interesting article by our Italian colleagues S. Migliore et al. found that 51.1% of their 92 RRMS patients with very mild clinical disability (EDSS ≤ 2.5) present cognitive dysfunction confirming previous reports, for example, [9] that cognitive impairment is only weakly correlated with physical disability. Furthermore, after subgrouping their RRMS patients by EDSS level, that is, EDSS ≤ 1.5 and EDSS 2 ≤ EDSS ≤ 2.5, they found a different pattern of impairment, with the less disabled group showing deficits in verbal memory and executive function and the more severe group having additional impairment in information processing speed and visual memory.

O. Ekmekci from Turkey provided us with a comprehensive review of the most recent clinical, neuropsychological, and neuroradiological features of pediatric-onset multiple sclerosis. In contrast to adult-onset MS, children diagnosed with MS are mostly impaired on the domains of attention, processing speed, visuomotor skills, intelligence, and language. Intriguing is the report that young age at disease onset appears to be the strongest risk factor for this impairment, implying the possibility that inflammatory demyelination and neurodegeneration may significantly impact the developing central nervous system (CNS) and neural networks. This is also evident on a pediatric MS patient's academic achievements and quality of life. The article also underlines the necessity of including cognitive screening and monitoring of cognitive impairment in the routine clinical practice of pediatric neurologists working with pediatric MS.

M.N. Mickens et al. from the USA, in an interesting study, examined the relationships between MS impairment, unmet family needs, and caregiver mental health. They provide us with a structural equation model demonstrating the mediational effect of unmet family needs (household, information, financial, social, support, and health) on the relationship between MS impairments (neurological, cognitive, behavioral, emotional, and functional) and caregiver mental health (satisfaction with life, anxiety, burden, and depression). They conclude that intervention research on MS caregivers in Latin America should consider focusing on caregiver mental health problems by addressing unmet family needs and teaching the caregiver's ways to deal with MS patient impairments. These findings may have implications not only for Latin American caregivers but also for European and potentially MS caregivers in any part of the world.

Shifting from impact to rehabilitation of cognitive impairment in MS, L. Messinis et al. from Greece provide an interesting article on a multicenter randomized controlled trial to assess the efficacy of computer-assisted cognitive rehabilitation in RRMS patients. They included fifty-eight clinically stable RRMS patients that were randomized to receive either computer-assisted (RehaCom) functional cognitive training with an emphasis on episodic memory, information processing speed/attention, and executive functions for 10 weeks (IG; *n* = 32) or standard clinical care (CG; *n* = 26). They found that only the IG group showed significant improvements in verbal and visuospatial episodic memory, processing speed/attention, and executive functioning from pre- to postassessment. Moreover, the improvement obtained on attention was retained over 6 months providing evidence on the long-term benefits of this type of intervention. Treated patients also rated the intervention positively and were more confident about their cognitive abilities following treatment. The study provides evidence regarding the positive impact of functional cognitive training with ecologically valid computerized cognitive tasks in a Greek sample of RRMS patients with moderate cognitive impairment severity and relatively low disability status. It also confirms findings noted by previous similar cognitive rehabilitation studies (see, e.g., [20, 21]).

C. Guijarro-Castro et al. from Spain provide an important article describing the research protocol of an innovative efficacy study on the impact of telestimulation or distance cognitive stimulation, with and without the support of face-to-face cognitive stimulation in MS patients with a disability level of EDSS ≤ 6. They stipulate that this novel research could help establish the usefulness of telematic cognitive stimulation (TCS) or, in its absence, face-to-face help for the alleviation of cognitive impairments in MS.

G. Nasios et al. from Greece provide an interesting overview on the most recent information and findings regarding the efficacy of repetitive transcranial magnetic stimulation (rTMS) in alleviating cognitive impairment in MS. The article stipulates that due to the lack of effective pharmacological treatments for cognition in MS, cognitive rehabilitation and other nonpharmacological interventions such as repetitive transcranial magnetic stimulation (rTMS) have recently emerged. The article focuses on the brain's functional reorganization in MS, theoretical and practical aspects of rTMS utilization in humans, and its potential therapeutic role in treating cognitively impaired MS patients.

From the contributing articles in this special issue, it becomes obvious that tremendous progress has been made in our understanding of the neuropsychological features of MS. That being said, guidelines on dealing or treating cognitive decline in MS are not yet available. The available disease-modifying treatments have shown minimum efficacy in alleviating cognitive impairment. Cognitive rehabilitation and other noninvasive brain stimulation techniques such as rTMS have shown reasonable effectiveness in certain clinical trials. To date, cognitive rehabilitation appears to be the current intervention of choice. The need for large-scale pharmaceutical and neurobehavioral interventions to clarify this issue, however, remains a priority.
